# Data Resource Profile Update: The Dutch National Intensive Care Evaluation (NICE) registry

**DOI:** 10.1093/ije/dyag088

**Published:** 2026-06-07

**Authors:** Ferishta Bakhshi-Raiez, Marie-José Roos-Blom, Dave A Dongelmans, Mark van den Boogaard, Bas C T van Bussel, Dylan W de Lange, Rob J Bosman, Roy van den Berg, Sesmu M Arbous, Jubi E de Haan, Jan Jaap Spijkstra, Dineke Benjamins, Lennart Krijgsman, Maxim H de Rooij, Michiel L Erkamp, Mart J de Graaff, Ruud A L de Waal, Nicolette F de Keizer, Sylvia Brinkman

**Affiliations:** National Intensive Care Evaluation (NICE) Foundation, Amsterdam, The Netherlands; Department of Medical Informatics, Amsterdam UMC, Location University of Amsterdam, Amsterdam, The Netherlands; Amsterdam Public Health, Quality of Care and Digital Health, Amsterdam, The Netherlands; National Intensive Care Evaluation (NICE) Foundation, Amsterdam, The Netherlands; Department of Medical Informatics, Amsterdam UMC, Location University of Amsterdam, Amsterdam, The Netherlands; Amsterdam Public Health, Quality of Care and Digital Health, Amsterdam, The Netherlands; National Intensive Care Evaluation (NICE) Foundation, Amsterdam, The Netherlands; Amsterdam Public Health, Quality of Care and Digital Health, Amsterdam, The Netherlands; Department of Intensive Care Medicine, Amsterdam UMC, University of Amsterdam, Amsterdam, The Netherlands; National Intensive Care Evaluation (NICE) Foundation, Amsterdam, The Netherlands; Department of Intensive Care Medicine, Radboud University Medical Center, Nijmegen, The Netherlands; National Intensive Care Evaluation (NICE) Foundation, Amsterdam, The Netherlands; Department of Intensive Care Medicine, Maastricht University Medical Center, Maastricht, The Netherlands; Cardiovascular Research Institute Maastricht, Maastricht University, Maastricht, The Netherlands; Care and Public Health Research Institute, Maastricht University, Maastricht, The Netherlands; National Intensive Care Evaluation (NICE) Foundation, Amsterdam, The Netherlands; Department of Intensive Care Medicine, University Medical Center Utrecht, University Utrecht, Utrecht, The Netherlands; National Intensive Care Evaluation (NICE) Foundation, Amsterdam, The Netherlands; Department of Intensive Care Medicine, OLVG, Amsterdam, The Netherlands; National Intensive Care Evaluation (NICE) Foundation, Amsterdam, The Netherlands; Department of Intensive Care Medicine, Elisabeth-TweeSteden Hospital, Tilburg, The Netherlands; National Intensive Care Evaluation (NICE) Foundation, Amsterdam, The Netherlands; Department of Intensive Care Medicine, Leiden University Medical Center, Leiden, The Netherlands; National Intensive Care Evaluation (NICE) Foundation, Amsterdam, The Netherlands; Department of Adult Intensive Care Medicine, Erasmus University Medical Center, Rotterdam, The Netherlands; National Intensive Care Evaluation (NICE) Foundation, Amsterdam, The Netherlands; Department of Intensive Care Medicine, Amsterdam UMC, University of Amsterdam, Amsterdam, The Netherlands; National Intensive Care Evaluation (NICE) Foundation, Amsterdam, The Netherlands; Department of Intensive Care Medicine, Saxenburgh Medical Center, Hardenberg, The Netherlands; National Intensive Care Evaluation (NICE) Foundation, Amsterdam, The Netherlands; Department of Intensive Care Medicine, Groene Hart Hospital, Gouda, The Netherlands; National Intensive Care Evaluation (NICE) Foundation, Amsterdam, The Netherlands; Department of Intensive Care Medicine, Elkerliek Hospital, Helmond, The Netherlands; National Intensive Care Evaluation (NICE) Foundation, Amsterdam, The Netherlands; Department of Intensive Care Medicine, Dijklander Hospital, Purmerend, The Netherlands; National Intensive Care Evaluation (NICE) Foundation, Amsterdam, The Netherlands; Department of Intensive Care Medicine, St. Antonius Hospital, Nieuwegein, The Netherlands; National Intensive Care Evaluation (NICE) Foundation, Amsterdam, The Netherlands; Department of Intensive Care Medicine, Amphia Hospital, Breda, The Netherlands; National Intensive Care Evaluation (NICE) Foundation, Amsterdam, The Netherlands; Department of Medical Informatics, Amsterdam UMC, Location University of Amsterdam, Amsterdam, The Netherlands; Amsterdam Public Health, Quality of Care and Digital Health, Amsterdam, The Netherlands; National Intensive Care Evaluation (NICE) Foundation, Amsterdam, The Netherlands; Department of Medical Informatics, Amsterdam UMC, Location University of Amsterdam, Amsterdam, The Netherlands; Amsterdam Public Health, Quality of Care and Digital Health, Amsterdam, The Netherlands

**Keywords:** quality registry, quality improvement, intensive care units, data collection, databases, benchmarking, data quality, critical care

Key Features

- The National Intensive Care Evaluation (NICE) foundation is a Dutch quality registry that was established in 1996 to monitor, assess, and improve the quality of care in adult intensive care units (ICUs).
- The registry currently includes all 81 Dutch ICU locations, with ∼75000 ICU admissions recorded annually. The database now contains >1.5 million admissions.
- The registry primarily consists of routinely collected clinical data during ICU stays. Data are submitted monthly (via comma-separated values (CSV) files or Microsoft Access) or daily (via Fast Healthcare Interoperability Resources). The Master DataSet is the core dataset including demographics, comorbidities, the Clinical Frailty Scale, and physiological and laboratory data from the first 24 hours, reason for ICU admission (Acute Physiology and Chronic Health Evaluation IV), and both ICU and in-hospital mortality. Annual organizational data are also collected per ICU, including hospital type, ICU capacity, staffing, and specialized functions. Extended registration domains cover topics such as mechanical ventilation, pain management, medication safety, sequential organ failure assessment, ICU bed availability, complications, and nursing activity.
- The registry follows patients until hospital discharge. Annual linkage with the national health insurance database (Vektis) enables long-term mortality tracking. Previous linkages include data from the Netherlands Emergency Department Evaluation Database and Statistics Netherlands.
- NICE welcomes collaboration on ICU care and outcomes research. For more information or data access, contact info@stichting-nice.nl.

## Data resource basics

Critically ill patients admitted to the intensive care unit (ICU) require continuous monitoring, life-sustaining treatments, and vital organ support. Due to their vulnerability and the intensity of care required, ICU patients contribute substantially to in-hospital mortality and healthcare costs. Monitoring outcomes and improving quality of care are essential to enhance recovery and ensure the efficient use of resources. The outcomes measured in this context provide valuable insights into the effectiveness of care and serve as a basis for evaluating and improving clinical practice. Comparing patient outcomes across hospitals (i.e. benchmarking) is a key strategy to identify areas for improvement. Without such comparisons, individual hospitals lack external references to objectively assess their performance. However, meaningful comparisons must account for variations in the case-mix across ICUs, which strongly influence outcomes such as mortality [1].

Recognizing the need for structured quality evaluation, a group of intensivists established the Dutch National Intensive Care Evaluation (NICE) foundation in 1996 to enable hospital benchmarking by systematically collecting standardized data on ICU admissions to identify unwarranted variation in outcomes and to support local and national quality-improvement efforts [2]. Reflecting the importance of multidisciplinary collaboration, the NICE board currently comprises 3 nurses alongside 12 intensivists. Over the past decade, the number of ICU locations in the Netherlands has declined due to the centralization of care and hospital mergers, in which intensive care services were often continued at a single site. Since 2016, the registry has provided nationwide coverage of all 81 mixed medical–surgical Dutch ICU locations. These include 8 ICUs located in an academic hospital, 28 ICUs located in a non-academic teaching hospital, and 45 ICUs located in a peripheral hospital. The ICUs are distributed across the Netherlands, with a higher concentration in the western urban region, as shown in Fig. 1. The registry is funded by these participating ICUs. The NICE registry includes data on >1 500  000 ICU admissions, with ∼75 000 new records per year [2].

**Figure:**
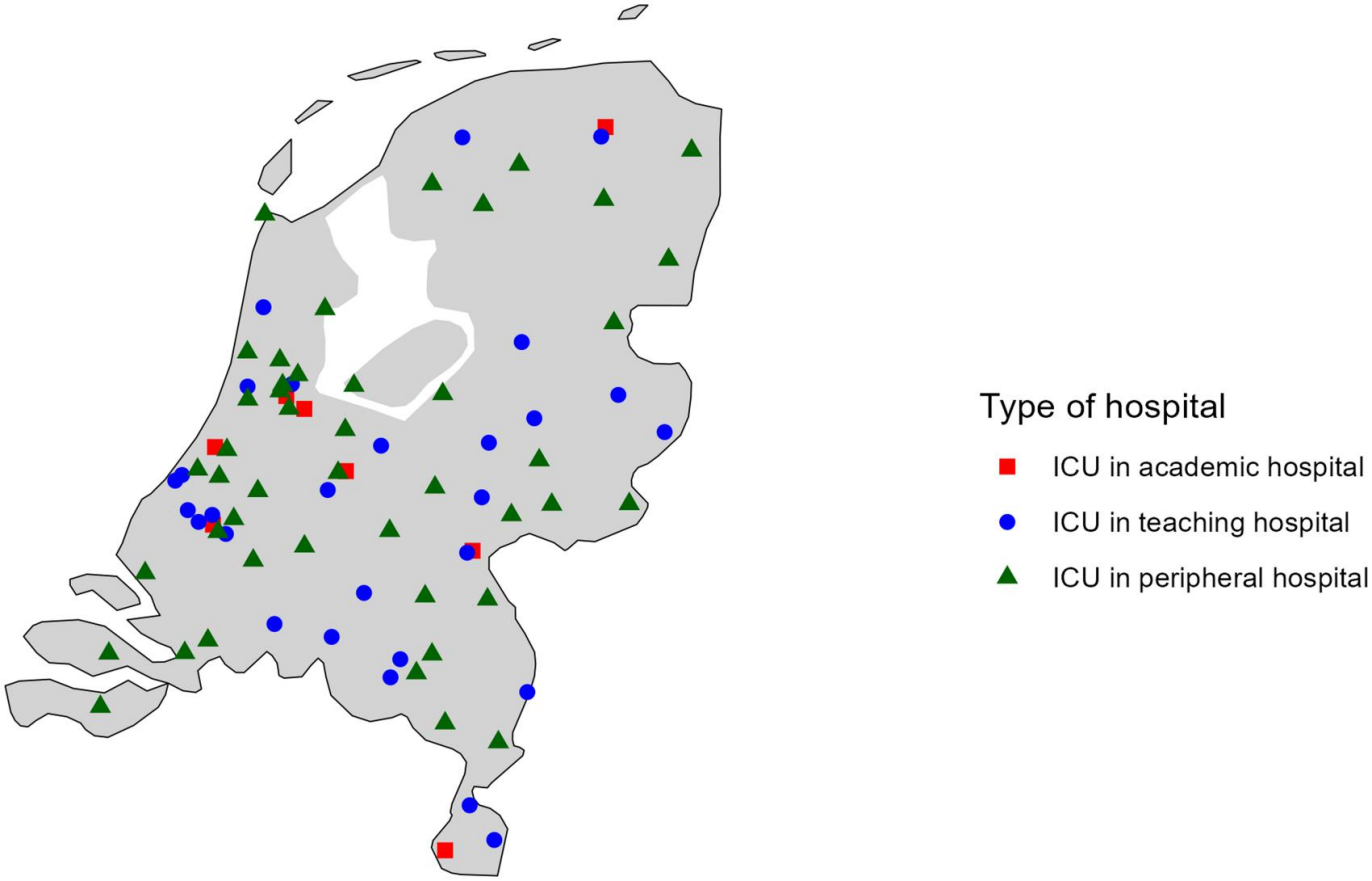
Figure 1 Distribution of the 81 ICU locations participating in the NICE registry in 2024.

The data in the NICE registry are derived from routine clinical practice via electronic health records (EHRs). Patient data are pseudonymized at the source and no direct identifiers are shared. Data can be securely uploaded monthly via Microsoft Access or comma-separated values (CSV) files, or provided daily through Health Level Seven Fast Healthcare Interoperability Resources (HL7-FHIR) messages. Microsoft Access submissions will be phased out in favor of HL7-FHIR, which enables faster, more automated, and less labor-intensive data transfer, improving real-time availability and integration with hospital systems.

The Department of Medical Informatics at Amsterdam UMC is responsible for processing, analysing, and safeguarding the data of the NICE registry. Processes are certified under ISO 9001 (quality management) and ISO 27001 (information security management). The NICE registry operates under the oversight of the national Content Governance Committee (IGC), which assesses the content of the data collection, and the national Data Governance Committee (DGC), which monitors and audits data processing and compliance. The NICE registry received a declaration from the Medical Ethics Review Committee of Amsterdam UMC (reference 2023.0259), confirming that the study does not fall under the Dutch Medical Research Involving Human Subjects Act (in Dutch: “Wet medisch-wetenschappelijk onderzoek met mensen” or WMO). Under the General Data Protection Regulation, a waiver of informed consent applies due to the acute and short-term nature of ICU admissions.

To gain more insight into the total patient trajectory or to enhance research opportunities without increasing the administrative burden for ICUs, the NICE registry has been linked to external data sources, either on a recurring or one-time basis, depending on the purpose and feasibility.

## Data collected

The NICE registry collects routinely recorded secondary healthcare data. All ICUs provide a core dataset (i.e. the Master DataSet or MDS) with demographics, comorbidities, Clinical Frailty Scale, physiological and laboratory data from the first 24 hours of ICU admission, reason for ICU admission based on the Acute Physiology and Chronic Health Evaluation (APACHE) IV classification [3], and both ICU and in-hospital mortality for each ICU admission. The NICE registry calculates severity scores and mortality risks based on several prognostic models, including the Simplified Acute Physiology Score II [4], APACHE II [5], and APACHE IV [3]. Although the performance of these models is similar [6], for research purposes, the APACHE IV model is preferred due to its broader choice in diagnostic categories. Table 1 presents characteristics of the 206 814 ICU admissions between 1 January 2022 and 1 January 2025, stratified by APACHE IV inclusion criteria [3]. The APACHE IV model excludes patients <16 years old, ICU stays of <4 hours, hospital stays of >365 days, transplant recipients (except for hepatic and renal transplants), burn patients, and patients with missing APACHE IV diagnoses, admission types, or discharge dates. Only the first ICU admission was included for readmitted patients.

**Table 1:** Demographics of ICU admissions between 1 January 2022 and 1 January 2025.

	Total ICU population	Fulfilling APACHE IV model inclusion criteria^a^
Number of admissions	206 814	190 104
Age [mean (SD)]	62.3 (16.0)	62.4 (16.0)
Male [*N* (%)]	127 104 (61.5)	117 012 (61.6)
Admission type [*N* (%)]		
Medical	113 778 (55.0)	102 575 (54.0)
Urgent surgery	24 244 (11.7)	21 463 (11.3)
Elective surgery	68 188 (33.0)	66 066 (34.8)
Mechanical ventilation on ICU admission [*N* (%)]	86 003 (41.6)	79 829 (42.0)
Mechanical ventilated in first 24 hours of ICU admission [*N* (%)]	100 131 (48.4)	92 406 (48.6)
Chronic comorbidities [*N* (%)]		
Renal insufficiency	10 201 (4.9)	9139 (4.8)
Respiratory insufficiency	26 979 (13.0)	24 520 (12.9)
Cardiovascular insufficiency	7875 (3.8)	7010 (3.7)
Malignancy	12 210 (5.9)	11 017 (5.8)
Immunological insufficiency	18 824 (9.1)	16 667 (8.8)
Cirrhosis	3203 (1.5)	2828 (1.5)
Diabetes	34 815 (16.8)	32 109 (16.9)
APACHE III Acute Physiology Score [mean (SD)]	44.9 (25.9)	44.6 (25.7)
APACHE III Score [mean (SD)]	56.8 (27.8)	56.5 (27.6)
APACHE IV mortality risk [mean (SD)]	–	16.1 (22.1)
Length of ICU stay (24-hour periods) [median (IQR)]	1.1 (0.8–2.8)	1.1 (0.8–2.7)
ICU mortality [*N* (%)]	19 518 (9.4)	16 552 (8.7)
Hospital mortality [*N* (%)]	26 640 (12.9)	22 748 (12.0)

a APACHE IV exclusion criteria: age <16 years, ICU stay <4 hours, hospital stay >365 days, transplant (except hepatic/renal), burns, missing APACHE IV diagnosis, admission type, or discharge date; only first ICU admission per hospitalization is included.

IQR, interquartile range.

Each ICU also submits annual organizational data, such as hospital type (academic, teaching, or peripheral), number of ICU beds, specialized functions (e.g. trauma unit), and staff numbers. ICUs may also participate in additional registration domains covering different topics. Table 2 lists all registration domains, quality indicators, and their added value. It also shows the initiation year of each registration domain and indicates that new domains have been introduced since the previous Data Resource Profile of the NICE registry in 2015 [2]. All registered variables and definitions are described in detail in the NICE data dictionary, available online [7]. The ICU Registration Committee is a multidisciplinary committee supporting the NICE Foundation in fulfilling its mission, among others by annually evaluating the quality indicators and underlying data for meaningfulness, feasibility, accuracy, and relevance, and by considering new items as needed. Only routinely recorded, automatically extractable healthcare (of EHRs) data are collected to minimize administrative burden and indicators must support continuous ICU care improvement. Many quality indicators have improved over time, also showing reduced variation between ICUs, indicating that benchmarking and continuous feedback contribute to more consistent and higher-quality care across Dutch ICUs [8].

**Table 2:** Overview of the NICE registration domains.

Initiation year	Registration domain	Description	Current quality indicators	Added value and importance
1996	MDS	Core dataset including demographics, physiology, laboratory results, chronic comorbidities, Clinical Frailty Scale, reasons for ICU admission, and clinical outcome data for each ICU admission	Percentage of ventilated ICU patients Length of ICU and hospital stay ICU and hospital mortality Standardized Mortality Ratio (SMR) Number of ICU readmissions (total, within 24 or 48 hours)	Enables fair comparison of outcomes by adjusting for case-mix differences across ICUs or stratification for process indicators. Allows benchmarking and quality-improvement initiatives based on reliable, standardized data
2000	Sequential Organ Failure Assessment (SOFA)	Daily recording of the degree of failure across six organ systems (respiratory, coagulation, renal, cardiovascular, liver, and neurological)	Number of patients with SOFA score of >10 Percentage of patients with decreasing/increasing SOFA trend Percentage of patients receiving renal replacement therapy Number of days on renal replacement therapy	Supports evidence-based decision-making by identifying trends in daily organ function. Helps assess the effectiveness of interventions and provides critical data for research and protocol development
2006	Quality Indicators Intensive Care (QIIC)	Includes: number of operational ICU beds and number of ICU nurses present per shift per day, and the start and end time of each mechanical ventilation period	Ventilation duration Average occupancy rate Days with 100% occupancy Nurse-to-patient ratio	Optimizes resource utilization (ICU efficiency) and ensures adequate staffing to minimize errors and prevent adverse events. Promotes safer patient environments and better patient outcomes
2006	Complications	Includes information on complications that occurred during ICU stay documented per day at patient level, including pneumothorax (developed in the ICU), re-intubation, and line sepsis	Percentage of patients with line sepsis Percentage of patients with re-intubation Percentage of patients with a pneumothorax	Provides data to refine clinical protocols, enabling targeted prevention strategies. Facilitates early identification of complications, reducing morbidity and mortality rates. Supports benchmarking across ICUs to identify best practices and drive continuous improvement
2015	Patient acuity registration	Includes a variety of nursing interventions per shift at the patient level (e.g. monitoring vital signs, administering medications, wound care, and mobilization) to calculate among others the Nursing Activity Score, reflecting nursing care intensity	Average care intensity per patient and per FTE nurse	Provides insight into the nursing workload and ensures optimal nurse allocation based on patient needs, identifying potential staffing gaps to enhance patient safety and prevent ICU nurse burnout. Promotes efficient care delivery while minimizing operational inefficiencies
2016	NICE2Improve pain	Includes process information on each pain measurement	Percentage of patients with execution of pain assessments each shift Percentage of patients with acceptable pain scores Percentage of patients with timely reassessment of pain in case of high pain scores Percentage of patients with timely normalization of high pain scores	Enables care teams to optimize pain-management processes through a user-friendly dashboard on pain scores, supporting the full PDCA cycle and stimulating timely interventions, ultimately leading to improved pain control and higher patient satisfaction
2023	NICE2Improve delirium	Includes information on the delirium screening instrument used and the presence of delirium	Percentage of patients screened on delirium Percentage of patients without delirium Number of delirium-free days per 100 patient days *(in development)* Average duration of delirium in days *(in development)*	Reduces the risk of delirium by identifying high-risk patients early through a user-friendly dashboard, supporting the full PDCA cycle to enhance preventive care strategies
2023	NICE2Improve pressure ulcers	Includes information on the presence of pressure ulcers per ICU admission	Percentage of patients screened for pressure ulcers Percentage of patients without pressure ulcers	Reduces the risk of pressure ulcers by identifying high-risk patients through a user-friendly dashboard, supporting the full PDCA cycle to enhance preventive care strategies
2023	NICE2Improve ventilation	Includes process information on each mechanical ventilation episode, such as tidal volume, ventilation mode, and blood gases	Percentage of patients with application of appropriate tidal volume in oxygenation disorders Percentage of patients with application of appropriate tidal volume without oxygenation disorders Percentage of patients with supportive ventilation after 48 hours	Enhances ventilation practices by providing detailed data on ventilation duration and applying tidal volume through a user-friendly dashboard, supporting the full PDCA cycle and enabling targeted adjustments, which ultimately improve respiratory care
2025	NICE2Improve antibiotic *(in development)*	Includes process information on prescribed antibiotics and lab tests	Percentage of patients with therapeutic drug monitoring Percentage of patients with application of SDD or SOD Percentage of patients with SDD or SOD and collection of surveillance cultures Resistance evaluation Days of therapy	Optimizes antibiotic management by providing accurate data on culture collection and therapeutic drug monitoring through a user-friendly dashboard, supporting the full PDCA cycle to reduce resistance prevalence and improve treatment effectiveness
2025	NICE2Improve blood *(in development)*	Includes process information on blood transfusions and lab results	Percentage of of unnecessary red blood cell transfusions Percentage of patients with omission of necessary red blood cell transfusions Percentage of patients with timely red blood cell transfusion Percentage of patients with no unnecessary plasma transfusions	Promotes more efficient blood management by minimizing unnecessary transfusions and ensuring timely administration of necessary transfusions through a user-friendly dashboard, supporting the full PDCA cycle to improve patient safety and reduce costs

FTE, full-time equivalent; PDCA, Plan-Do-Check-Act; SOD, Selective Oropharyngeal Decontamination; SOFA, Sequential Organ Failure Assessment; SDD, Selective Digestive Decontamination.

To ensure reliable data and valid benchmarking, continuous monitoring of data quality is crucial. Before submitting data, each ICU must send two representatives—typically intensivists or ICU nurses—for mandatory training on variable definitions and registration rules. ICUs are encouraged to periodically repeat this training, which is offered four times a year. ICUs are further supported by an online data dictionary, e-learning modules, an extensive frequently asked questions (FAQ) section, and direct contact via email or phone. Each ICU admission record undergoes >600 automated quality checks, assessing extreme values, logical inconsistencies, and completeness, before being added to the registry. ICUs receive detailed feedback reports for data validation and correction. Additionally, on-site validations are conducted to verify the completeness and correctness of the submitted data. Data from ICUs with insufficient data quality are excluded from analyses and benchmarking until improvements are made and re-evaluated.

To support quality improvement, NICE provides feedback reports, online tools, dashboards, meetings, as well as on-demand and site-specific support. Biannual feedback reports allow ICUs to monitor progress and benchmark their performance. The online NICE Analysis Tool enables ICUs to conduct data analyses and gain deeper insights into their ICU performance. In addition, NICE2Share facilitates secure data sharing within collaboration networks, regional partnerships, and other cooperative initiatives via the Analysis Tool. The online NICE2Improve dashboard and a toolbox containing practical improvement strategies support ICUs to initiate and implement quality-improvement activities [9]. The public “Data in Beeld” website offers key ICU statistics for patients, families, and other interested parties [10]. Finally, an annual national conference provides a confidential setting for sharing and discussing results, enabling participants to clarify variations in outcomes and identify opportunities for quality improvement. On-demand support is available for specific questions or assistance in launching quality-improvement projects.

Beyond the standard indicators available within the NICE registry, which include outcomes up to hospital discharge, the registry has also been linked to external data sources to enhance research and insight into the total patient journey without increasing administrative burden. A major enhancement is the annual linkage with the national health insurance database Vektis, allowing analyses on long-term survival after ICU admission [11]. Since the previous Data Resource Profile of the NICE registry in 2015 [2], the registry has expanded its data linkages. Linkages used for research purposes with the Netherlands Emergency department Evaluation Database (NEED) and Statistics Netherlands (CBS) have enabled studies on, for example, the impact of emergency department length of stay and socioeconomic factors on ICU outcomes [12, 13]. While mortality remains a core outcome, assessing post-ICU quality of life is equally important. To address this, NICE collaborates with MONITOR-IC to collect patient-reported outcome measures (PROMs) for a better understanding of long-term recovery [14]. Ongoing efforts explore more systematic integration of these datasets and additional national quality registries or databases, such as the Dutch Hospital Data.

## Data resource use

The NICE registry is extensively used for quality assessment, research, and policy support. To limit the administrative burden, the dataset focuses on routinely recorded clinical variables, while ensuring high-quality, interoperable data through standardization with the Observational Medical Outcomes Partnership (OMOP) Common Data Model (CDM) and underlying vocabularies [15].

Furthermore, NICE data are used in national initiatives such as pandemic preparedness by the Dutch National Institute for Public Health (RIVM) [16]. During the COVID-19 pandemic, the NICE registry became a primary data provider for RIVM, government agencies, and hospitals [17]. Nationally, NICE is a key driver behind initiatives such as the Collaborating Quality Registries (SKR) and Collaborating Data Processors (SDV), streamlining quality registries in the Netherlands in response to evolving requirements from the IGC and the DGC [13]. Internationally, NICE contributes data to the Linking of Global Intensive Care (LOGIC) platform, enabling benchmarking across 18 countries and 1500 ICUs [18], and collaborates with ICU registries in other countries, such as the Intensive Care National Audit & Research Centre [19], the Australian and New Zealand Intensive Care Society [20], and the Critical Care Asia Africa network [21].

As the registry has expanded in scope and coverage since the previous Data Resource Profile of the NICE registry in 2015 [2], further valuable research has been enabled. Examples include evaluating process-oriented interventions through actionable quality indicators, assessing patient acuity and ICU nurse workload, and facilitating easier and more accessible regional ICU comparisons to monitor and improve quality of care. Since its establishment, data from NICE have supported >200 scientific publications and 16 PhD dissertations, contributing to critical care and quality improvement. NICE research includes clinical epidemiological research and registry-based research. Clinical epidemiological research examines outcome associations [22, 23], guideline validation [24–26], trend monitoring [27], and performance assessment [8]. Registry-based research focuses on audit and feedback [9, 28], case-mix models [11, 12], and optimizing data collection [29, 30]. All publications based on NICE data are listed on the NICE website [31].

## Strengths and weaknesses

The NICE registry covers all admissions to all Dutch adult ICUs, enabling comprehensive benchmarking, quality monitoring and improving, and research. Initiated by and for ICU professionals, NICE ensures both clinical relevance and ongoing innovation within the field of intensive care. The use of international standards facilitates data exchange and reuse for scientific research, public health monitoring, and policy development.

Nonetheless, the registry also faces several limitations. One constraint is limited data granularity, as only the most extreme laboratory and physiological values are collected, which restricts certain in-depth analyses using advanced machine-learning techniques. NICE is addressing this by collecting more granular data, including complete medication and laboratory data at least at an hourly level. Daily data exchange is not yet standard in all participating ICUs. However, an increasing number of ICUs are transitioning to data exchange via FHIR and its broader adoption is expected to significantly enhance the speed and effectiveness of quality-improvement initiatives, as well as operational management tasks such as staff planning and the surveillance of severe acute respiratory infections. A further limitation is the current lack of pre-ICU and post-ICU information. While single linkages with NEED (quality registry for emergency care) and MONITOR-IC (quality of life of ICU survivors) have been performed for specific research projects, systematic integration of these data into the NICE registry is still under exploration.

In addition, some registration domains are not yet widely adopted, limiting the national generalizability of insights derived from those domains. The NICE aims to increase adoption by promoting standardization and targeted training, offering implementation support, and strengthening national collaboration with professional associations to emphasize the clinical and scientific value of these data.

Since the previous Data Resource Profile of the NICE registry in 2015 [2], we have, among other initiatives, focused on supporting ICUs in actively improving care. With NICE2Improve, we emphasize more on actionable process quality indicators and provide a toolbox with concrete guidance and suggested action plans to help ICUs implement improvement actions in practice. With NICE2Share, we facilitate (regional) collaborations to compare data, learn from each other, and drive improvements. These efforts will continue, while future plans will additionally address the integration of PROMs and the broader adoption of more daily and granular data exchange, further enhancing support for quality improvement and shared decision-making.

The upcoming amendment to the Healthcare Quality, Complaints, and Disputes Act (WKKGZ), effective per 1 January 2026, provides a legal framework for pseudonymized data processing. With this law, participation by all Dutch hospitals is mandatory. This law also strengthens governance and standardization of the general quality registry landscape. NICE is actively implementing the requirements set by the IGC and DGC to ensure continued high-quality, compliant, and sustainable ICU data collection without administrative burden [32].

Finally, NICE continues to play a leading role in the responsible reuse of clinical data in line with European and national frameworks such as the European Health Data Space and the Dutch Integral Healthcare Agreement (IZA). With its mature infrastructure, validated data, and ongoing innovation efforts, the NICE registry is well positioned to remain a cornerstone of quality monitoring, benchmarking, and research in Dutch ICUs.

## Data resource access

Participants of the NICE registry can request access to (anonymized) data and analysis support through a standardized procedure outlined on the NICE website [33]. Research proposals are evaluated by the NICE Scientific Committee based on relevance, feasibility, and scientific merit.

The data are not publicly available due to privacy regulations. Opportunities for academic collaboration are available, particularly for projects that align with NICE’s mission to improve the quality of ICU care. Requests from external researchers must include background information, a clear research question, and a research plan. Data are not available for commercial use.

The use of a standardized data dictionary, which is available online [7], ensures uniformity and clear definitions, facilitating the reproducibility of research and enabling other researchers to replicate or build upon studies using NICE data. No proprietary software is required for basic access; standard statistical or database tools such as R and Python can be used for analysis. Currently, research is ongoing to assess the OMOP-CDM for federated analyses, potentially expanding future research access [34]. Transparency is further supported by making all publications based on NICE data publicly available on the NICE website [31]. Questions about data access or collaboration opportunities can be sent to info@stichting-nice.nl.

## Ethics approval

Not applicable. This article describes the structure and data-collection procedures of the NICE registry and does not involve human-subjects research as defined under applicable regulations.

## Data Availability

The data are not publicly available due to privacy regulations. Questions about data access or collaboration opportunities can be sent to info@stichting-nice.nl.
